# Characterization of Metabolite and Lipid Compositions in Lycopene-Enriched Egg Yolk Using Liquid Chromatography Quadrupole Time-of-Flight Mass Spectrometry

**DOI:** 10.3390/foods15101653

**Published:** 2026-05-09

**Authors:** Xianyu You, Jishi Wang, Zeying He, Xiaoxue Yu, Xin Zhao, Liuan Li, Chaoqi Ren

**Affiliations:** 1 Key Laboratory of Intelligent Breeding, Ministry of Agriculture and Rural Affairs (Ministry-Province Joint Establishment), Tianjin Key Laboratory of Agricultural Animal Breeding and Health Breeding, College of Animal Science and Veterinary Medicine, Tianjin Agricultural University, Tianjin 300392, China; xianyuyou0224@163.com (X.Y.);; 2 Key Laboratory for Environmental Factors Control of Agro-Product Quality Safety, Agro-Environmental Protection Institute, Ministry of Agriculture and Rural Affairs, Tianjin 300191, China

**Keywords:** lycopene-enriched egg yolk, UPLC-QTOF-MS, untargeted metabolomics, lipidomics

## Abstract

This study established an untargeted metabolomic approach based on ultra-performance liquid chromatography/quadrupole time-of-flight mass spectrometry (UPLC-QTOF-MS) to investigate differences in metabolites and lipid composition of lycopene-enriched egg yolk at different enrichment levels and conventional egg yolks. Principal component analysis and hierarchical clustering revealed clear unsupervised discrimination and separation among the control group and two treatment groups. Metabolomic analysis identified 14 differential metabolites, including amino acids, which were associated with 13 metabolic pathways such as cysteine and methionine metabolism. Lipidomic analysis revealed 48 significantly altered lipids, including phospholipids and glycerides. The results demonstrated that lycopene supplementation significantly altered the metabolic and lipid profiles of egg yolks. Specifically, lycopene enrichment upregulated phospholipid synthesis and increased the levels of antioxidant-related metabolites. This study confirms that untargeted metabolomics and lipidomics can effectively identify potential biomarkers in egg yolks with varying lycopene enrichment levels, offering new insights and a scientific basis for nutritional research and metabolic mechanism analysis of functional eggs.

## 1. Introduction

Eggs are widely favored by consumers because of their high nutritional value, affordability, and rich content of essential nutrients. Their nutritional value is partly attributed to the presence of bioactive lipids, which exert important biological activities and are found predominantly in egg yolks [1]. In recent years, with increasing consumer interest in health-promoting foods, demand has gradually shifted from conventional eggs to functional eggs enriched with specific nutrients or bioactive compounds [2]. Functional eggs are generally defined as eggs whose nutritional composition has been improved through dietary regulation, resulting in increased levels of beneficial nutrients and/or reduced levels of undesirable components, thereby providing added health benefits for consumers. For example, the global fortified eggs market was estimated at USD 409.9 million in 2024 and is projected to reach USD 648.2 million by 2030, while the broader functional foods market is also expanding rapidly worldwide [3,4,5]. Functional “specialty” eggs such as selenium-enriched eggs and folic acid-fortified eggs are expected to remain dominant in the global food market.

Lycopene, a type of carotenoid, is widely present in ripe red fruits, such as tomatoes, carrots, and watermelons. It plays a vital role in preventing cardiovascular and cerebrovascular diseases, enhancing immunity, exerting anti-inflammatory and anti-cancer effects, as well as exhibiting antioxidant properties and delaying aging [6,7]. The human body cannot synthesize lycopene on its own and relies solely on dietary intake for supplementation. Lycopene is safe for daily dietary supplementation, as no adverse health effects occur even at a daily intake as high as 75 mg/day [8]. In the field of poultry farming, the effects of lycopene on poultry production performance have been verified to some extent. An et al. reported that dietary lycopene supplementation in Hy-Line Brown laying hens for 28 consecutive days improved egg weight and laying rate [9]. Wang et al. showed that lycopene addition to broiler diets promoted average daily weight gain [10]. Shevchenko et al. demonstrated that dietary supplementation with different levels of lycopene in Hy-Line Brown laying hens improved egg quality, increased carotenoid levels in fresh eggs, and enhanced yolk color [11].

Metabolomics, as a vital branch of systems biology, reveals the relationship between metabolites and specific conditions through quantitative analysis of small-molecule metabolites within organisms. This approach identifies changes in metabolite levels across different states and, in the process, pinpoints differentially expressed metabolites [12]. Metabolic changes induced by disease diagnosis, drug research and development [13], and microbial research [14] have seen widespread use of this technology in their exploration. Within the scope of food testing, metabolomics holds great significance for key issues such as origin tracing [15], meat quality testing [10], dairy product testing [16], variety identification [17], processing technology [18], and quality and safety control [19].

Ultra-performance liquid chromatography (UPLC) is utilized in lipidomics for the separation of lipid components, followed by the application of tandem mass spectrometry (MS/MS) for identification. This enables the identification and quantitative analysis of hundreds of lipids [20]. Unlike metabolomics, lipidomics has a more concentrated research scope, focusing exclusively on lipid molecules among metabolites. It is also widely utilized in various research fields, encompassing medical disease diagnosis [21], cell membrane structure [22], signal transduction [23], and biological evolution [24]. Within food testing, lipidomics serves for food fraud identification [25], lipid characterization [20], and production time assessment [26]. Whether metabolomics or lipidomics, their final goal lies in finding metabolite biomarkers that can distinguish sample groups and interpreting the functional mechanisms of underlying biological processes.

Nowadays, attention to functional eggs is rising. However, research remains relatively scarce on core questions such as whether egg yolks with varying lycopene enrichment levels exhibit differences in metabolic profiles and lipid composition, how non-targeted omics technologies can be employed to identify their specific biomarkers, and how to elucidate the molecular mechanisms by which lycopene regulates egg yolk nutrition and metabolism. This study established ultra-performance liquid chromatography/quadrupole time-of-flight mass spectrometry (UPLC-Q-TOF) based untargeted metabolomics and lipidomics approaches, coupled with multivariate statistics, to analyze metabolic characteristics and lipid changes in egg yolks of hens supplemented with varying lycopene doses. Principal component analysis (PCA) was applied for three-group sample discrimination without prior knowledge, and an (Orthogonal Partial Least Squares Discriminant Analysis) OPLS-DA model was built to screen potential biomarkers and develop a discriminant model for egg yolks with different lycopene enrichment levels. Differential metabolite biological significance was further clarified via metabolic pathway analysis. This study has identified the mechanism by which lycopene regulates yolk nutrition between lipids and metabolism, offering new perspectives for food nutrition research and a basis for understanding related metabolic mechanisms.

## 2. Materials and Methods

### 2.1. Chemicals and Reagents

Methanol, acetonitrile, and isopropanol were all HPLC-grade reagents, purchased from Fisher Scientific (Fair Lawn, NJ, USA). HPLC-grade dichloromethane was obtained from Merck (Darmstadt, Germany), while formic acid and ammonium formate were sourced from Sigma-Aldrich (Darmstadt, Germany). Experimental water was purified using a Millipore Milli-Q system (Billerica, MA, USA), and lycopene standard (purity > 98%) was purchased from Shanghai Aladdin Biochemical Technology (Shanghai, China).

### 2.2. Egg Sample

Eggs with lycopene enrichment were from a prior experiment [27] (Test S1). The experiment included a control group (without lycopene), G60 (60 mg/kg diet lycopene), and G240 (240 mg/kg diet lycopene). On day 30, samples were collected, and 6 eggs per group were randomly chosen as biological replicates. Yolks were separated from albumen, pre-preserved at −80 °C overnight, vacuum freeze-dried for 72 h, ground to fine powder, and stored at −80 °C for further analysis. The lycopene concentration data for the enriched eggs used in this study were obtained from our previous experiments and have been reported in a published study [27] and in another manuscript accepted for publication. The average lycopene concentrations were 0.472 μg/mL in the G60 group and 0.340 μg/mL in the G240 group. Ethical approval was obtained for this experiment (Approval No.: 2024LLSC51).

### 2.3. Sample Preparation

Metabolomic sample extraction was based on previous studies [28], with minor modifications. Exactly 50 mg of lyophilized egg yolk powder was weighed and transferred to a 2 mL centrifuge tube, followed by the addition of 1 mL pre-cooled dichloromethane and methanol extract (30:10, *v*/*v*). The mixture was vigorously vortexed for a duration of 10 min. After each extraction, the sample was centrifuged at 8000 rpm at 4 °C for 10 min, and the supernatants were then combined. This extraction procedure was replicated three times. An amount of 1.2 mL of the combined solution was pipetted, evaporated overnight (12 h) in a fume hood, then reconstituted with 1.2 mL methanol, vortexed for 5 min, filtered through a 0.22 μm membrane. The diluted solution was stored at −80 °C and finally subjected to UPLC-QTOF analysis.

Lipidomic sample extraction was modified based on previous studies [29]. Exactly 100 mg of lyophilized egg yolk powder was weighed into a 15 mL centrifuge tube, and 6 mL isopropanol was added. The mixture was vortexed at 2000 rpm for 1 h. It was then centrifuged at 5000 rpm for 10 min under 4 °C conditions. After centrifugation, the supernatant was collected and filtered through a 0.22 μm membrane. The diluted solution derived from filtration was stored at −20 °C to support subsequent UPLC-QTOF analysis.

For metabolomic and lipidomic analyses, six biological replicates were included in each group. Throughout the entire analytical process, quality control (QC) samples and blanks were injected at an interval of three experimental samples. For instrumental analysis, the sample run order was rigorously randomized to eliminate biases caused by temporal drifts in instrumental sensitivity. Furthermore, during data processing, all datasets were normalized against QC samples to correct for experimental variations.

### 2.4. Uhplc-Q-Tof Untargeted Analysis

An Exion LC UHPLC system, in combination with a quadrupole-time-of-flight (Q-TOF) mass spectrometer (Triple TOF 6600, Sciex Inc., Foster City, CA, USA), was utilized for high-resolution mass analysis. Electrospray ionization (ESI) was carried out in both positive and negative modes, with the source voltages set at 5500 V and −4500 V, respectively. The supplementary ESI parameters were as follows: temperature, 500 °C; nebulizing gas (GS1) pressure, 50 psi; heater gas (GS2) pressure, 50 psi; and curtain gas (CUR) pressure, 35 psi. Full-scan time-of-flight mass spectrometry (TOF-MS) data were obtained within the *m*/*z* range of 70–1250, and independent data acquisition (IDA) was implemented to gather tandem mass spectrometry (MS/MS) spectra in the *m*/*z* range of 50–1250 during a single analytical run. In the full-scan TOF-MS experiment, the declustering potential (DP) and collision energy (CE) were set at 60 V and 10 eV, respectively. For the triggered MS/MS acquisition, the criteria encompassed an intensity threshold of 100 counts per second (cps), a mass tolerance of 50 millidalton, and a collision energy of 35 ± 15 eV (varied through collision energy spread).

An HSS T3 C18 column (2.1 × 100 mm, 1.8 μm; Waters, Milford, MA, USA) was employed for chromatographic separation. For metabolomics, column temperature was kept constant at 50 °C, flow rate set to 0.3 mL/min, and injection volume at 2 μL. Positive ion mode used mobile phases: (A) 0.1% formic acid in water and (B) methanol. Negative ion mode used mobile phase A: 5 mM ammonium formate in water (pH 7.5, adjusted with ammonia) and mobile phase B: methanol. Both modes adopted a 21 min gradient elution: 3% B (0–1.5 min), increased to 80% B at 11 min, raised to 97% B at 14 min, maintained until 18 min, then decreased to 3% B in 0.1 min, and re-equilibration was achieved after 2.9 min.

For lipidomics, the column temperature was maintained at 55 °C, with a flow rate of 0.26 mL/min and an injection volume of 2 μL. In positive ion mode, the mobile phases were (A) acetonitrile/water (60:40, *v*/*v*) and (B) isopropanol/acetonitrile (90:10, *v*/*v*). In negative ion mode, mobile phase A was acetonitrile/water (60:40, *v*/*v*) (pH 7.5, adjusted with ammonia), and mobile phase B was isopropanol/acetonitrile (90:10, *v*/*v*) (pH 7.5, adjusted with ammonia). A 27.5 min gradient elution program was used for both modes: At 1.5 min, the proportion of B increased from 30% to 54%; by 4 min, it rose to 55%; at 14 min, it further increased to 80%, then gradually reached 85% at 17 min. The proportion of B hit 100% at 17.5 min and was maintained until 21.5 min, followed by a decrease to 30% within 0.5 min, and re-equilibration was achieved after 5.5 min.

### 2.5. Data Processing and Statistical Analyses

Data processing was executed using Sciex OS software 3.0 (Sciex Inc., Foster City, CA, USA) for peak selection and annotation. MetaboAnalyst 6.0 was utilized for data pre-processing and multivariate statistical analysis. Metabolites with missing values exceeding 50% were excluded, and the remaining missing values were replaced with half of the minimum positive value, which is close to the limit of detection. Additionally, metabolites exhibiting a relative standard deviation (RSD) greater than 30% in QC samples were removed. The retained data were normalized through mean-centering and Pareto scaling (division by the square root of the standard deviation of each variable). Data acquired from both positive and negative ion modes were integrated into a single dataset for subsequent analysis. Multivariate analytical techniques, such as PCA and OPLS-DA, were employed to explore group separations and metabolic patterns. Differential metabolites were evaluated based on fold change (FC was calculated based on the normalized relative abundance of each metabolite or lipid as the ratio of the mean signal intensity in one group to that in the other compared group) and Student’s *t*-test *p*-values, with multiple testing correction implemented via false discovery rate (FDR). Variables with an FDR-adjusted *p*-value < 0.05, FC > 1.5, and variable importance in projection (VIP) from the OPLS-DA model greater than 1 were considered potential biomarkers. Metabolite identification was conducted by matching against an in-house high-resolution MS/MS library containing over 1000 standard compounds. Compounds were identified based on accurate mass, isotope pattern similarity, and MS/MS spectral matching according to commonly used practices in high-resolution LC-MS-based untargeted metabolomics. Features with a mass error < 5 ppm, isotope similarity within 10%, and spectral matching score > 80 were considered high-confidence annotations, whereas features meeting the mass and isotope criteria but lacking reliable MS/MS spectral matching were classified as tentative annotations [30,31]. Lipids were identified using LipidView software (version 1.2; Sciex Inc.) based on retention time, characteristic ion fragments, and the number of acyl carbons or acyl double bonds. Hierarchical clustering analysis (HCA) was performed to visualize sample groupings and metabolite abundance patterns under different conditions. Pathway and enrichment analysis were carried out using MetaboAnalyst with reference to the KEGG database.

## 3. Results and Discussion

### 3.1. Untargeted Metabolomics Analysis

**Figure 1:**
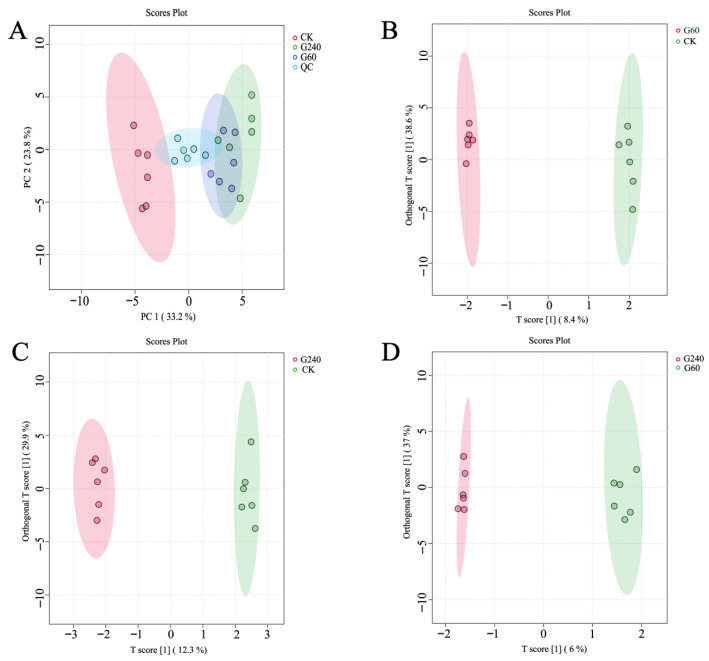
The PCA score plot (**A**) and OPLS-DA score plots ((**B**): G60 vs. CK; (**C**): G240 vs. CK; (**D**): G240 vs. G60) based on the metabolic profiles of egg yolk under different lycopene enrichment. The colored ellipses represent the 95% confidence regions for each group. (CK, control group; G60, group supplemented with 60 mg lycopene per kg of feed; G240, group supplemented with 240 mg lycopene per kg of feed; QC, quality control sample. CK-1 to CK-6, G60-1 to G60-6, and G240-1 to G240-6 represent the six biological replicates in the CK, G60, and G240 groups, respectively).

Metabolite profiling data from the control, G60, and G240 groups were analyzed by PCA based on UPLC-Q-TOF-MS data. After excluding metabolites with relative standard deviation (RSD) values > 30% in the QC samples, a total of 517 metabolites were retained for subsequent analysis. PCA was first performed to detect potential outliers and to provide an initial assessment of group discrimination. The PCA score plot showed that all samples were located within the 95% confidence interval, indicating that no obvious outliers were present. In addition, the QC samples were tightly clustered near the origin, demonstrating good analytical stability and reproducibility (Figure 1A). A clear separation was observed between the control group and the lycopene-enriched groups (G60 and G240). PC1 and PC2 explained 33.2% and 23.8% of the total variance, respectively, indicating that the score plot was representative of the overall variation in the dataset (Figure 1A). To further distinguish metabolic differences among the groups, supervised OPLS-DA was performed. The OPLS-DA model for the G60 versus control comparison is shown in Figure 1B. The corresponding model parameters (R2Y = 71.8% and Q2 = 23.5%) indicated acceptable explanatory and predictive ability without obvious overfitting. Although the R2X value of the primary predictive component was relatively low (8.38%), the orthogonal component contributed additional explanatory power (R2X = 38.6%, Q2 = 38.9%), suggesting that the model remained generally interpretable and usable (Figure S1A). Similarly, valid models were obtained for the other pairwise comparisons. The G240 versus control model showed good performance (R2X = 12.3%, R2Y = 87.1%, Q2(cum) = 50.6%; Figure S1B), while the G240 versus G60 model was comparatively weaker but still acceptable (R2X = 11.9%, R2Y = 86.2%, Q2(cum) = 27.0%; Figure S1C). In addition, permutation tests with 1000 iterations confirmed that all OPLS-DA models were statistically significant and not overfitted (*p* < 0.05; Supplementary Figure S2). Overall, these results demonstrate that the constructed OPLS-DA models were reliable and suitable for subsequent differential metabolite analysis.

### 3.2. Identification of Potential Biomarkers

To comprehensively analyze the chemical differences in egg yolks under different lycopene enrichment levels, differential metabolites were screened with the criteria: *p*-value < 0.05, FC > 1.5, and VIP > 1. This process identified 14 significant metabolites. The concentration range of downregulated metabolites was 0.66–0.11-fold among the groups, whereas the range of upregulated metabolites was 1.50–1.84-fold. Among these, 7 were unequivocally identified by matching to high-resolution MS/MS databases, while the remaining 7 were tentatively identified based on high-resolution MS/MS spectral matching. Their extracted ion chromatograms, isotopic distributions, and product ion matches are provided in Figure S4. These metabolites were categorized into several classes, including four amino acids and their derivatives, two sterols, two vitamin compounds, one carotenoid, one peptide, one sugar derivative, one ketone compound, and two other compounds (Table 1). Comprehensive details are listed in Supplementary Table S1.

**Figure 2:**
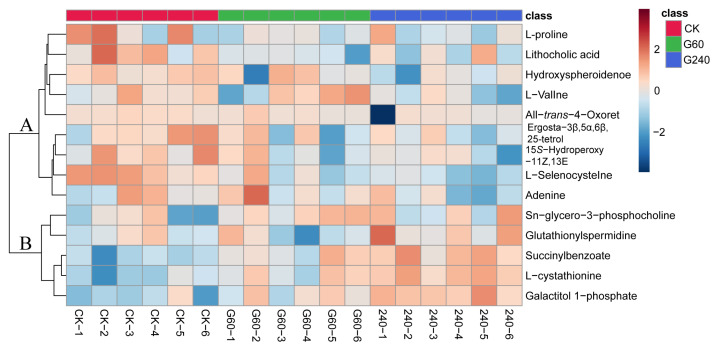
Heatmap of identified metabolites with significant changes among CK, G60, and G240 egg yolk. (CK, control group; G60, group supplemented with 60 mg lycopene per kg of feed; G240, group supplemented with 240 mg lycopene per kg of feed. CK-1 to CK-6, G60-1 to G60-6, and G240-1 to G240-6 represent the six biological replicates in the CK, G60, and G240 groups, respectively.).

Following the identification of the 14 differential markers, the outcomes were visualized in the heatmap (Figure 2). The *x*- and *y*-axes represent the 18 individual samples and the identified metabolites, respectively. Color intensity corresponds to the relative abundance of each metabolite. The heatmap illustrates the clustering relationships among the 18 samples through HCA. This method groups samples with comparable metabolic characteristics into separate clusters without any prior knowledge of the group identities. The selected 14 metabolites identified were clustered into two distinct regions in the heatmap. Region A and region B contained 9 and 5 metabolites, respectively. Metabolites in region A exhibited the highest abundance in the control group. Specifically, ergosta-3β,5α,6β,25-tetrol, 15*S*-hydroperoxy-11Z,13E, lithocholic acid, L-selenocysteinewere, hydroxyspheroidenone and L-proline were more abundant in the control group compared to G60 and G240 samples. Notably, lithocholic acid and hydroxyspheroidenone displayed the most significant difference, with fold changes ranging from 0.44 to 0.11. In contrast, adenine and all-*trans*-4-oxoretinoic acid showed similar levels in the control group and G60 samples, both higher than those in G240. Conversely, metabolites in region B were more abundant in G60 and G240 than in the control group. Moreover, the richness of the G240 group is higher than that of the G60 group. These results indicate that enrichment with different lycopene enrichment levels exerts a significant effect on the contents of egg yolk metabolites.

### 3.3. Effects of Different Lycopene Enrichment Levels on Egg Yolk Metabolic Pathways

**Figure 3:**
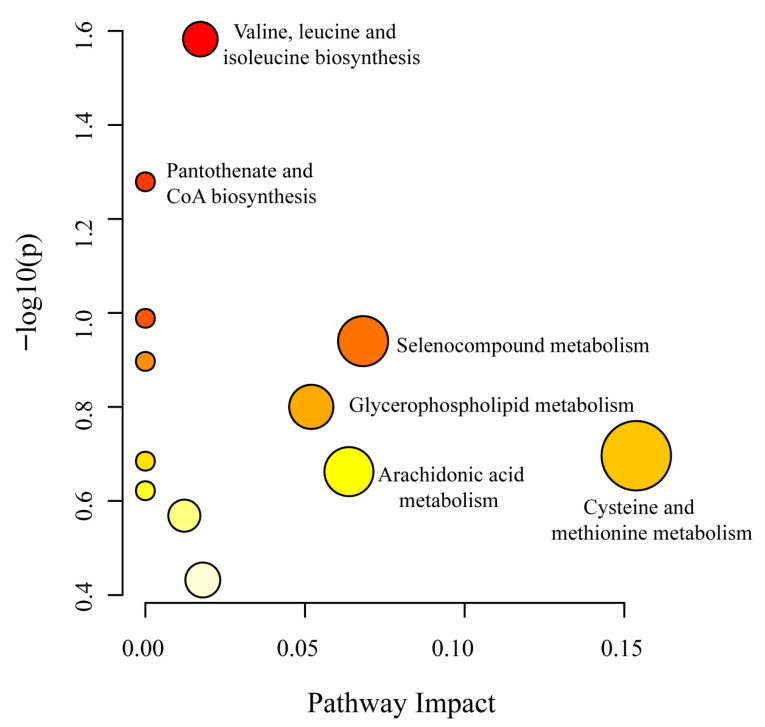
The matched pathways according to the *p*-values from the pathway enrichment analysis and pathway impact values from the pathway topology analysis.

**Figure 4:**
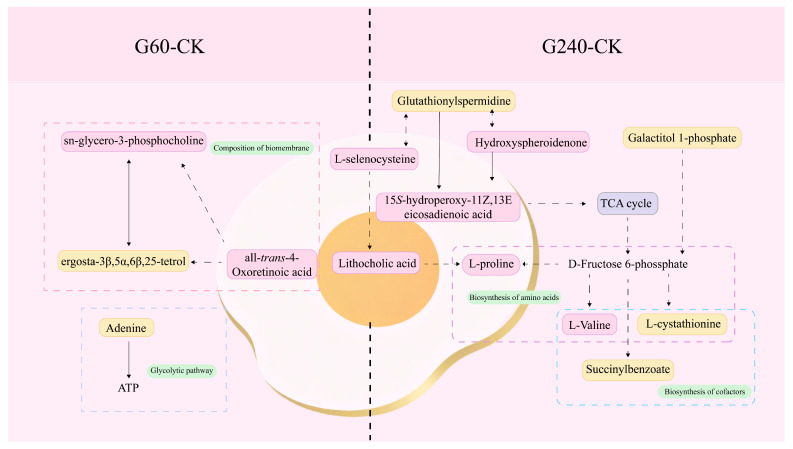
Altered metabolic pathways and related metabolites in egg yolk under different levels of lycopene enrichment. (Pink square: down-regulation; yellow square: up-regulation. The five substances on the egg surface indicated metabolites that changed under both enrichment levels. CK, control group; G60, group supplemented with 60 mg lycopene per kg of feed; G240, group supplemented with 240 mg lycopene per kg of feed). Solid arrows indicate direct effects, and dashed arrows indicate indirect effects.

Metabolic pathway analysis of differentially expressed metabolites was performed using MetaboAnalyst 6.0 to elucidate key metabolic pathways associated with the response of egg yolks to lycopene at varying enrichment levels. The analysis revealed significant alterations in 13 metabolic pathways, with 4 pathways exhibiting impact values exceeding 0.05. (Figure 3 and Table S2). As illustrated in Figure 3 and Figure 4, the differentially expressed metabolites identified were mapped to multiple biologically relevant pathways based on the KEGG pathway database, with many metabolites participating in diverse metabolic processes. The outcomes indicate variations exist between egg yolks with differing levels of lycopene enrichment. Moreover, egg yolks enriched with lycopene exhibited more pronounced differences compared to the control group, with these variations being closely correlated to the degree of lycopene enrichment. The primary metabolic pathways involved include, cysteine and methionine metabolism, arachidonic acid metabolism, selenocompound metabolism, glycerophospholipid metabolism and the biosynthesis of other metabolites. These pathways were mainly associated with the differential abundance of metabolites such as L-cystathionine, L-selenocysteine, 15*S*-hydroperoxy-11Z,13E-eicosadienoic acid, and sn-glycero-3-phosphocholine. Together, these results suggest that lycopene enrichment may affect amino acid metabolism, antioxidant-related pathways, fatty acid metabolism, and phospholipid metabolism in egg yolk.

### 3.4. Effects of Different Lycopene Enrichment Levels on Potential Biomarkers of the Metabolome in Egg Yolk

For clarity, the differential metabolites are discussed below according to their metabolic characteristics and potential biological relevance, including carotenoid-related compounds, bile acid and sterol-related metabolites, amino acid-related metabolites, and other metabolites associated with fatty acid, vitamin, phospholipid, and carbohydrate metabolism.

Among the carotenoid-related compounds, hydroxyspheroidenone was identified as the most significantly altered metabolite, with a substantial downregulation in G240 (FC: 0.11 under G240), while little change was observed in the G60 group (FC: 1.01 under G60). Since both hydroxyspheroidenone and lycopene are carotenoids, this pattern may indicate a potential interaction between carotenoid compounds under high lycopene supplementation. Previous studies have suggested that excessive dietary carotenoid supplementation in laying hens may suppress the deposition of other carotenoids in egg yolk [32,33,34]. Consequently, when hydroxyspheroidenone faces substantial competition from lycopene, its content exhibits a markedly significant reduction trend. Supplementing with lower levels of carotenoids increases their concentration, yet this effect is suppressed at higher supplementation levels [33]. This study explains why lycopene enrichment levels in the G240 group were lower than those in the G60 group in our previous experiments.

Among bile acid- and sterol-related metabolites, lithocholic acid was identified as the second significantly altered metabolite, exhibiting marked downregulation in both G60 and G240 treatments (FC: 0.44 under G240; FC: 0.30 under G60). As a secondary bile acid, lithocholic acid is known to exhibit hepatotoxicity at elevated levels [35]. Given the reported antioxidant and hepatoprotective effects of lycopene, the reduced lithocholic acid level observed here may reflect an altered hepatic metabolic state associated with lycopene supplementation [36]. In addition, ergosta-3β,5α,6β,25-tetrol, a sterol-related metabolite, was also reduced in the enriched groups. This finding suggests that lycopene enrichment may affect sterol- and cholesterol-related metabolism in egg yolk, which is consistent with previous reports that lycopene supplementation can influence sterol transport and lipid metabolism in laying hens [37].

Among the amino acid-related metabolites, L-valine and L-proline both showed downregulation in the lycopene-enriched groups. These amino acids are involved in protein metabolism, muscle synthesis, and physiological regulation [38,39]. Yang et al. [40] reported that dietary lycopene supplementation in chickens could affect amino acid metabolism, including L-proline. In contrast, L-cystathionine was upregulated in the G240 group. As an intermediate in methionine metabolism, this metabolite may indicate that high-dose lycopene supplementation influenced sulfur amino acid metabolism and related antioxidant responses. L-selenocysteine, the major biologically active form of selenium in organisms [41,42], is closely related to selenium metabolism and antioxidant defense [43]. Its decrease in the lycopene-treated groups may suggest a shift in antioxidant-related metabolic balance under lycopene enrichment [44].

Several other metabolites also showed notable alterations. The 15*S*-Hydroperoxy-11Z,13E-eicosadienoic acid is a product of fatty acid metabolism. Its relatively reduced content may be associated with lycopene’s effect of significantly delaying protein degradation [45]. All-*trans*-4-oxoretinoic acid participates in the metabolic processes of vitamin A. It has been demonstrated that increasing the dosage of lycopene supplements leads to heightened vitamin A deposition [46], resulting in reduced levels of vitamin A metabolites. In contrast, glutathionylspermidine was increased, particularly in the G240-related comparisons, which may reflect enhanced antioxidant-related metabolic activity. Sn-glycero-3-phosphocholine, a key intermediate in phospholipid metabolism and lecithin biosynthesis, showed increased abundance, suggesting that lycopene supplementation may influence phospholipid turnover in egg yolk and thereby contribute to changes in yolk physicochemical and nutritional properties. In addition, adenine, succinylbenzoate, and galactitol 1-phosphate may reflect alterations in nucleotide metabolism, quinone-related metabolism, and carbohydrate metabolism, respectively. Galactitol 1-phosphate as an intermediate metabolite in galactose metabolism, may influence carbohydrate metabolism alongside increased lycopene accumulation levels [47], thereby promoting the synthesis of glycoproteins and glycolipids. However, because direct evidence linking these metabolites to egg functional quality remains limited, these interpretations should be considered preliminary.

### 3.5. Effects of Different Lycopene Enrichment Levels on Potential Biomarkers of Lipid Metabolism in Egg Yolk

**Figure 5:**
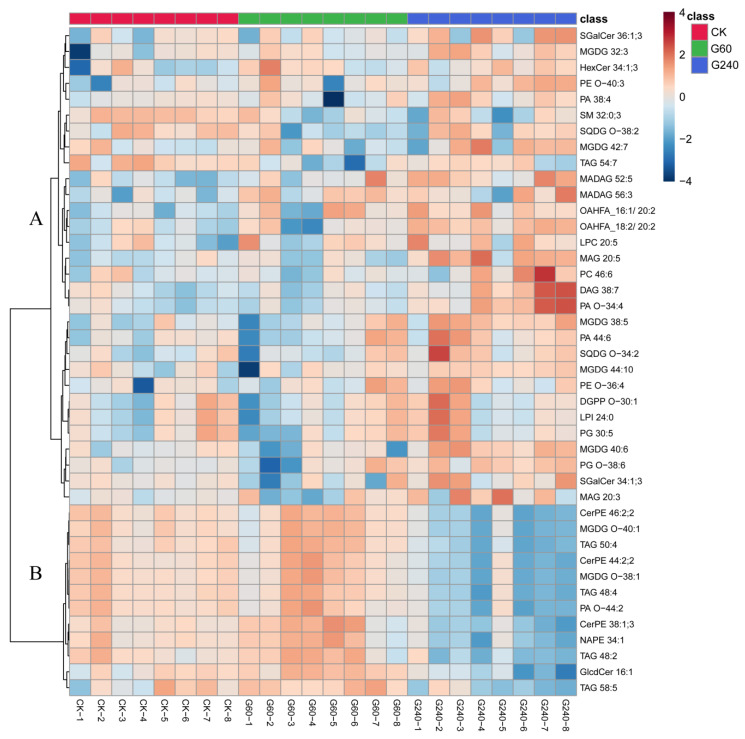
Heatmap of identified lipid with significant changes among CK, G60, and G240 egg yolk. (CK, control group; G60, group supplemented with 60 mg lycopene per kg of feed; G240, group supplemented with 240 mg lycopene per kg of feed. CK-1 to CK-6, G60-1 to G60-6, and G240-1 to G240-6 represent the six biological replicates in the CK, G60, and G240 groups, respectively).

Lipidomic data were analyzed by PCA following metabolomic analytical methodologies, and significant differences were observed among experimental groups (Figure S5). The clustering distribution of QC samples indicates that this analytical method has high reliability. A total of 48 differential lipids were identified via pairwise comparisons between groups, including 15 phospholipids, 11 glycerides, 9 glycolipids, 9 sphingolipids, 2 ceramides, and 2 fatty acids. The content heatmap of these lipids (Figure 5) and the FC, *p*, and VIP values comparing the control group with G60 and G240 (Table 2) demonstrate significant differences in egg yolk lipids among different lycopene enrichment levels and the control group. Compared with the control group, only 2 lipids in G60 showed differential changes. In contrast, G240 exhibited significant differences from the control group, with 34 lipids undergoing remarkable alterations, among which 22 were upregulated. Comprehensive analysis of metabolomic and lipidomic data revealed that the effect of dietary lycopene supplementation on laying hens was much stronger at 240 mg/kg than at 60 mg/kg, indicating that lycopene has a significant impact on egg yolks and that its influence on lipids is greater than that on metabolites. Notably, studies indicate that the human body absorbs 2.12 times more carotenoids from eggs than from vegetables [48]. Thus, the scientific community widely agrees that animal-derived foods like eggs contain more readily absorbable lycopene than plant-based alternatives. This finding provides a theoretical foundation for developing lycopene-enriched eggs.

Egg yolks have a high lipid content, accounting for approximately 33%, and contain nearly all types of lipids [49]. Lipids in egg yolks not only serve as a source of nutrition but also possess multiple functions such as physiological regulation and maintaining cell membranes. Consumers of functional eggs pay greater attention to their nutritional value. Previous experiments have shown that lycopene supplementation can significantly increase the Haugh unit by 10%, improve the laying rate by approximately 5%, increase egg weight, and deepen the egg yolk color.

Phospholipids regulate cholesterol and triglyceride transport while maintaining membrane integrity and normal fluidity. In this study, no significant alterations were observed in any phospholipids within the G60 group. Conversely, in the G240 group, nearly all phospholipids exhibited significant upregulation except for PA O-44:2 and PG 46:3, with PG 46:3 showing approximately ~10-fold downregulation compared to the control group. Research indicates that high-dose lycopene activates PPARα, leading to an overall upregulation of the phospholipid synthesis pathway [50]. Glycerolipids play a role in providing energy and nutrition. In this study, all glycerolipids in the G240 group were upregulated, with DAG 38:7 showing a ~2.5-fold increase relative to the control group, excluding triglycerides (TAG). As the biosynthetic pathways of triglycerides and phospholipids both originate from glycerol-3-phosphate, enhanced phospholipid synthesis results in reduced triglyceride levels. Glycerolipids in eggs serve to assist glycerolipids and phospholipids. Sphingolipids can synergize with phospholipids to construct membrane structures in embryonic cells and neural tissues. Ceramides function as intermediate metabolites of sphingolipids. There were no significant differences in sphingolipids and ceramides between the G60 group. Within the G240 group, only α-Galactosylceramide (SGalCer) and hexosylceramide (HexCer) showed an increase, while all others exhibited a downward trend. SGalCer and HexCer may be influenced by high concentrations of lycopene, participating in specific pathway regulation. The downregulation of sphingolipids indicates that the egg maintains a low level of oxidative stress [51], providing a good explanation for the oxidative stress-relieving effect of lycopene. The composition of fatty acids directly influences the nutritional value and edible quality of egg yolks. A moderate increase in fatty acid content may be regarded to some extent as a positive indicator of lipid metabolism adjusting towards a healthier state. In this study, the addition of lycopene resulted in predominantly downregulated metabolomics profiles and largely upregulated lipidomics profiles in eggs, indicating that lycopene enrichment significantly enhances the nutritional composition and antioxidant capacity of eggs.

## 4. Conclusions

This study established an untargeted metabolomics and lipidomics approach based on UPLC-Q-TOF to systematically investigate the effects of different lycopene enrichment levels on the metabolic profiles and lipid compositions of egg yolks. Lycopene enrichment significantly altered both the metabolite profile and lipid composition of egg yolks, with lipid changes being more pronounced. The G60 and G240 treatments collectively affected 14 metabolites and 48 lipids, involving amino acids and their derivatives, sterols, carotenoids, phospholipids, glycerides, and glycolipids, as well as metabolic pathways such as cysteine and methionine metabolism and arachidonic acid metabolism. These findings suggest that lycopene enrichment may influence antioxidant-associated metabolism, phospholipid metabolism, lipid composition, and carotenoid-related metabolic interactions in egg yolk. In particular, metabolites such as hydroxyspheroidenone, L-cystathionine, L-selenocysteine, and sn-glycero-3-phosphocholine may serve as candidate biomarkers of lycopene enrichment. This study fills a gap in research concerning the metabolic and lipid changes in lycopene-enriched egg yolks, enabling the precise identification of candidate biomarkers in yolks across a gradient of lycopene supplementation levels, and providing novel insights into food nutrition research.

## Figures and Tables

**Table 1:** Fold changes, *p*-values, and VIP values of metabolites in different groups.

Compound	G60 vs. CK				G240 vs. CK				G240 vs. G60			
	FC	*p* Values	VIP	Up or Down	FC	*p* Values	VIP	Up or Down	FC	*p* Values	VIP	Up or Down
Lithocholic acid ^a^	0.30	2.55 × 10^−3^	1.61	↓↓	0.44	2.68 × 10^−2^	1.29	↓↓	1.45	1.14 × 10^−1^	0.82	-
L-proline ^a^	0.54	6.58 × 10^−3^	1.06	↓	0.59	2.11 × 10^−2^	0.96	↓	1.10	3.68 × 10^−1^	0.49	-
15*S*-hydroperoxy-11Z,13E eicosadienoic acid	0.56	3.92 × 10^−4^	1.18	↓	0.58	9.59 × 10^−4^	1.14	↓	1.03	8.47 × 10^−1^	0.70	-
L-selenocysteine	0.59	4.06 × 10^−5^	1.15	↓	0.57	3.05 × 10^−5^	1.22	↓	0.98	4.93 × 10^−1^	0.21	-
ergosta-3β,5α,6β,25-tetrol	0.66	6.67 × 10^−6^	1.06	↓	0.70	2.66 × 10^−6^	1.00	-	1.06	2.24 × 10^−1^	0.45	-
All-*trans*-4-Oxoretinoic acid	0.66	1.29 × 10^−5^	1.02	↓	0.57	1.09 × 10^−1^	1.17	↓	0.84	3.38 × 10^−1^	1.23	-
L-Valine ^a^	0.74	5.10 × 10^−2^	0.73	-	0.62	1.12 × 10^−4^	1.10	↓	0.85	4.84 × 10^−1^	0.44	-
Adenine ^a^	1.11	1.18 × 10^−1^	1.05	-	1.06	2.52 × 10^−1^	0.64	-	1.55	6.80 × 10^−3^	1.92	↑
L-cystathionine ^a^	1.23	3.30 × 10^−2^	1.82	-	1.55	4.32 × 10^−4^	2.55	↑	1.25	3.74 × 10^−2^	2.17	-
Glutathionylspermidine	1.12	3.53 × 10^−1^	0.63	-	1.84	8.45 × 10^−5^	2.49	↑	1.63	8.71 × 10^−4^	2.20	↑
Hydroxyspheroidenone	1.01	5.33 × 10^−1^	0.77	-	0.11	6.77 × 10^−8^	5.35	↓↓	0.11	2.70 × 10^−4^	4.67	↓↓
Sn-glycero-3-phosphocholine	1.55	2.69 × 10^−2^	2.89	↑	1.43	1.69 × 10^−1^	1.47	-	0.93	4.97 × 10^−1^	1.09	-
Succinylbenzoate ^a^	1.23	3.79 × 10^−2^	1.79	-	1.56	6.20 × 10^−4^	2.55	↑	1.26	3.28 × 10^−2^	2.23	-
Galactitol 1-phosphate	1.22	5.36 × 10^−2^	1.69	-	1.54	3.95 × 10^−4^	2.55	↑	1.26	1.66 × 10^−2^	2.36	-

FC > 1.5, VIP > 1 and *p* < 0.05 indicate significant up-regulation, FC < 0.67, VIP > 1 and *p* < 0.05 indicate significant down-regulation. ↑: up-regulation (1.5 < FC < 2.0), ↓: down-regulation (0.5 < FC < 0.67), ↓↓: down-regulation (FC < 0.5). (CK, control group; G60, group supplemented with 60 mg lycopene per kg of feed; G240, group supplemented with 240 mg lycopene per kg of feed.). ^a^ identified using MS/MS high-resolution library.

**Table 2:** Fold changes, *p*-values, and VIP values of lipids in different groups.

Compound	G60 vs. CK				G240 vs. CK				G240 vs. G60			
	FC	*p* Values	VIP	Up or Down	FC	*p* Values	VIP	Up or Down	FC	*p* Values	VIP	Up or Down
PA O-34:4	0.97	8.46 × 10^−1^	0.24	-	2.43	1.08 × 10^−4^	2.23	↑↑	2.51	4.59 × 10^−5^	2.41	↑↑
PC 46:6	0.91	3.29 × 10^−1^	0.85	-	1.68	7.34 × 10^−3^	1.47	↑	1.85	2.40 × 10^−3^	1.81	↑
PG O-38:6	0.96	4.93 × 10^−1^	0.80	-	1.62	9.55 × 10^−5^	1.70	↑	1.70	8.69 × 10^−3^	1.72	↑
PA 44:6	0.96	5.40 × 10^−1^	0.55	-	1.57	5.26 × 10^−4^	1.58	↑	1.64	3.88 × 10^−3^	1.55	↑
NAPE 34:1	1.11	5.19 × 10^−1^	0.58	-	0.70	1.11 × 10^−2^	1.37	-	0.63	9.72 × 10^−3^	1.49	↓
PG 30:5	0.87	3.68 × 10^−1^	0.89	-	1.36	2.52 × 10^−2^	1.17	-	1.56	5.93 × 10^−3^	1.46	↑
PA 38:4	0.90	2.55 × 10^−1^	0.94	-	1.39	3.11 × 10^−5^	1.43	-	1.56	3.22 × 10^−3^	1.58	↑
LPI 24:0	0.89	4.55 × 10^−1^	0.70	-	1.35	3.22 × 10^−2^	1.12	-	1.51	8.02 × 10^−3^	1.34	↑
DGPP O-30:1	0.91	5.31 × 10^−1^	0.58	-	1.37	2.38 × 10^−2^	1.18	-	1.50	6.77 × 10^−3^	1.34	↑
LPC 20:5	1.18	2.17 × 10^−1^	1.06	-	1.71	9.98 × 10^−4^	1.70	↑	1.44	1.50 × 10^−2^	1.34	-
PA O-44:2	1.01	8.51 × 10^−1^	0.10	-	0.61	5.73 × 10^−3^	1.70	↓	0.60	1.74 × 10^−2^	1.57	↓
PE O-40:3	1.10	4.94 × 10^−1^	0.70	-	1.64	6.75 × 10^−4^	1.70	↑	1.49	3.86 × 10^−3^	1.54	-
PE O-36:4	1.11	4.62 × 10^−1^	0.63	-	1.52	4.50 × 10^−3^	1.51	↑	1.37	1.08 × 10^−2^	1.18	-
PG 46:3-	0.93	8.81 × 10^−1^	0.14	-	0.11	4.98 × 10^−6^	3.80	↓↓	0.12	8.08 × 10^−6^	4.07	↓↓
LPC 18:2-	1.17	2.32 × 10^−1^	0.89	-	1.88	3.24 × 10^−4^	2.5	↑	1.60	5.59 × 10^−3^	1.60	↑
DAG 38:7	0.91	6.49 × 10^−1^	0.54	-	2.46	1.16 × 10^−3^	2.10	↑↑	2.69	3.22 × 10^−4^	2.41	↑↑
TAG 48:2	1.16	4.70 × 10^−1^	0.77	-	0.61	8.83 × 10^−3^	1.62	↓	0.53	5.63 × 10^−3^	1.79	↓
MAG 20:5	0.96	5.39 × 10^−1^	0.45	-	1.77	2.95 × 10^−6^	1.96	↑	1.85	1.58 × 10^−6^	2.04	↑
TAG 58:5	1.23	1.16 × 10^−1^	1.66	-	0.68	8.35 × 10^−2^	1.09	-	0.55	8.73 × 10^−4^	2.04	↓
TAG 50:4	1.07	7.49 × 10^−1^	0.35	-	0.60	1.47 × 10^−3^	1.70	↓	0.56	2.90 × 10^−3^	1.78	↓
TAG 48:4	1.02	9.72 × 10^−1^	0.15	-	0.59	4.02 × 10^−3^	1.76	↓	0.58	7.49 × 10^−3^	1.72	↓
TAG 54:7	0.69	5.38 × 10^−3^	2.57	-	1.07	4.68 × 10^−1^	0.34	-	1.56	1.91 × 10^−3^	1.57	↑
MADAG 56:3	1.46	1.16 × 10^−2^	2.41	-	1.80	1.20 × 10^−2^	1.54	↑	1.23	4.00 × 10^−1^	0.51	-
MAG 20:3	0.93	3.75 × 10^−1^	1.02	-	1.75	1.06 × 10^−2^	1.47	↑	1.89	1.18 × 10^−2^	1.78	↑
MADAG 52:5	1.09	1.61 × 10^−1^	0.84	-	1.53	7.45 × 10^−7^	1.67	↑	1.41	1.26 × 10^−5^	1.50	-
TAG 52:2-	0.68	2.49 × 10^−1^	1.00	-	0.37	1.65 × 10^−3^	2.30	↓	0.54	3.99 × 10^−5^	2.19	↓
SQDG O-38:2	0.66	2.77 × 10^−3^	2.60	↓	1.11	5.57 × 10^−1^	0.33	-	1.68	3.79 × 10^−3^	1.59	↑
MGDG 40:6	0.80	1.15 × 10^−1^	1.63	-	1.64	1.03 × 10^−4^	1.74	↑	2.04	1.05 × 10^−4^	2.13	↑↑
MGDG O-38:1	1.05	9.28 × 10^−1^	1.13	-	0.58	3.00 × 10^−3^	1.79	↓	0.56	6.51 × 10^−3^	1.76	↓
MGDG O-40:1	1.08	6.69 × 10^−1^	0.42	-	0.65	3.75 × 10^−3^	1.56	-	0.60	5.73 × 10^−3^	1.64	↓
MGDG 38:5	1.01	8.58 × 10^−1^	0.06	-	1.68	1.84 × 10^−5^	1.81	↑	1.67	8.75 × 10^−4^	1.70	↑
SQDG O-34:2	0.94	5.74 × 10^−1^	0.48	-	1.52	1.33 × 10^−3^	1.51	↑	1.62	9.97 × 10^−4^	1.58	↑
MGDG 44:10	0.97	6.81 × 10^−1^	0.58	-	1.83	5.76 × 10^−3^	1.87	↑	1.88	4.02 × 10^−2^	1.79	↑
MGDG 42:7	0.90	3.37 × 10^−1^	0.80	-	1.42	2.92 × 10^−2^	1.15	-	1.58	6.96 × 10^−3^	1.46	↑
MGDG 32:3	1.08	3.66 × 10^−1^	0.98	-	1.61	1.71 × 10^−3^	1.65	↑	1.49	1.64 × 10^−5^	1.56	-
SM 32:0;3	0.66	2.52 × 10^−4^	2.88	↓	0.85	1.34 × 10^−1^	0.82	-	1.29	1.89 × 10^−1^	0.70	-
CerPE 44:2;2	1.07	7.85 × 10^−1^	0.30	-	0.59	4.55 × 10^−3^	1.73	↓	0.56	6.04 × 10^−3^	1.78	↓
SGalCer 36:1;3	0.95	7.11 × 10^−1^	0.20	-	1.62	1.52 × 10^−2^	1.43	↑	1.71	6.64 × 10^−3^	1.58	↑
GlcdCer 16:1	1.27	6.95 × 10^−2^	1.59	-	0.73	5.70 × 10^−2^	1.15	-	0.59	7.19 × 10^−3^	1.75	↓
SGalCer 34:1;3	0.93	9.73 × 10^−2^	1.45	-	1.39	4.82 × 10^−4^	1.33	-	1.68	1.39 × 10^−3^	1.74	↑
CerPE 46:2;2	1.04	8.64 × 10^−1^	0.18	-	0.63	3.27 × 10^−3^	1.62	↓	0.60	6.37 × 10^−3^	1.64	↓
HexCer 30:2;2	1.01	9.58 × 10^−1^	0.09	-	1.64	6.55 × 10^−5^	1.76	↑	1.62	4.81 × 10^−4^	1.63	↑
HexCer 34:1;3	1.16	1.03 × 10^−1^	1.35	-	1.53	6.30 × 10^−4^	1.52	↑	1.32	3.64 × 10^−3^	1.26	-
CerPE 38:1;3	1.20	2.09 × 10^−1^	1.11	-	0.75	2.14 × 10^−2^	1.16	-	0.62	8.76 × 10^−3^	1.49	↓
Cer 46:1;2	0.86	1.29 × 10^−1^	1.15	-	0.64	1.10 × 10^−3^	1.52	↓	0.75	1.08 × 10^−2^	1.20	-
Cer 40:2;2-	1.02	5.73 × 10^−1^	0.47	-	0.52	6.04 × 10^−2^	1.40	↓	0.52	3.85 × 10^−5^	2.32	↓
OAHFA_18:2/20:2	1.00	8.24 × 10^−1^	0.26	-	1.72	9.87 × 10^−8^	1.92	↑	1.73	5.30 × 10^−5^	1.92	↑
OAHFA_16:1/20:2	1.44	1.93 × 10^−1^	0.93	-	1.63	3.79 × 10^−6^	1.80	↑	1.42	1.27 × 10^−3^	1.45	-

FC > 1.5, VIP >1 and *p* < 0.05 indicate significant up-regulation, FC < 0.67, VIP > 1 and *p* < 0.05 indicate significant down-regulation. ↑: up-regulation (1.5 < FC< 2.0), ↑↑: up-regulation (FC > 2), ↓: down-regulation (0.5 < FC < 0.67), ↓↓: down-regulation (FC < 0.5). (CK, control group; G60, group supplemented with 60 mg lycopene per kg of feed; G240, group supplemented with 240 mg lycopene per kg of feed.) Abbreviations: PA, phosphatidic acid; PC, phosphatidylcholine; PG, phosphatidylglycerol; PE, phosphatidylethanolamine; LPC, lysophosphatidylcholine; LPI, lysophosphatidylinositol; NAPE, N-acyl phosphatidylethanolamine; DGPP, diacylglycerol pyrophosphate; DAG, diacylglycerol; TAG, triacylglycerol; MAG, monoacylglycerol; MADAG, monoacetyldiacylglycerol; MGDG, monogalactosyldiacylglycerol; SQDG, sulfoquinovosyldiacylglycerol; SM, sphingomyelin; Cer, ceramide; CerPE, ceramide phosphoethanolamine; HexCer, hexosylceramide; SGalCer, sulfatide/galactosylceramide; GlcdCer, glucosylceramide; OAHFA, (O-acyl)-omega-hydroxy fatty acid.

## Data Availability

The original contributions presented in this study are included in the article/Supplementary Material. Further inquiries can be directed to the corresponding authors.

## Supplementary Materials

The following supporting information can be downloaded at: https://www.mdpi.com/article/10.3390/foods15101653/s1, Test S1: Sample preparation; Table S1: Detailed information of identified metabolites; Table S2: Pathways affected by tebuconazole exposure; Figure S1: Multivariate statistical model performance evaluation for OPLS-DA models; Figure S2: Permutation plot for OPLS-DA models; Figure S3: The PCA score plots based on the lipidomic profiles in egg yolk under different lycopene enrichment; Figure S4: Extracted ion chromatogram, isotope pattern, fragmentation patterns analyzed and MS/MS spectrum against library of the identified metabolites; Figure S5: The PCA score plot based on the lipid profiles of egg yolk under different lycopene enrichment. The colored ellipses represent the 95% confidence regions for each group.
