# Supplementary material for: Characterization of Metabolite and Lipid Compositions in Lycopene-Enriched Egg Yolk Using Liquid Chromatography Quadrupole Time-of-Flight Mass Spectrometry
Source: Foods. 2026 May 9;15(10):1653. doi: 10.3390/foods15101653 (PMC13205705; doi:10.3390/foods15101653)
Supplement: Supplementary file 1 [file foods-15-01653-s001.zip › foods-4205432-supplementary.pdf]

Supplementary material for

Characterization of Metabolite and Lipid Compositions in lycopene-enriched egg yolk using liquid chromatography quadrupole time-of-flight mass spectrometry

Xianyu You[ab1], Jishi Wang[b1], Zeying He[b], Xiaoxue Yu[a], Xin Zhao[a], Liuan Li[a*], Chaoqi Ren[a*]

[a]Key Laboratory of Intelligent Breeding, Ministry of Agriculture and Rural Affairs (Ministry-Province Joint Establishment), Tianjin Key Laboratory of Agricultural Animal Breeding and Health Breeding, College of Animal Science and Veterinary Medicine, Tianjin Agricultural University, Tianjin 300392, P.R. China

[b]Key Laboratory for Environmental Factors Control of Agro-product Quality Safety, Ministry of Agriculture and Rural Affairs, Agro-Environmental Protection Institute, Ministry of Agriculture and Rural Affairs, Tianjin 300191, P.R. China.

*Corresponding author:

Chaoqi Ren, Key Laboratory of Intelligent Breeding, Ministry of Agriculture and Rural Affairs (Ministry-Province Joint Establishment), Tianjin Key Laboratory of Agricultural Animal Breeding and Health Breeding, College of Animal Science and Veterinary Medicine, Tianjin Agricultural University, Tianjin 300392, P.R. China;

Tel.: 18510000895;

E-mail: renchaoqi92@163.com

Liuan Li, Key Laboratory of Intelligent Breeding, Ministry of Agriculture and Rural Affairs (Ministry-Province Joint Establishment), Tianjin Key Laboratory of Agricultural Animal Breeding and Health Breeding, College of Animal Science and Veterinary Medicine, Tianjin Agricultural University, Tianjin 300392, P.R. China;

Tel.:13752365700;

E-mail: anliuli2003@163.com

[1]These authors contributed equally to this paper.

Enrichment of Lycopene in Egg Yolk

Test S1 Sample preparation

Egg samples for the experiment were obtained from Tianjin Jimuyuan Livestock and Poultry

Breeding Cooperative Base. A total of 180 330-day-old North China local chickens were selected and randomly divided into 3 groups, with 20 replicates per treatment and 3 chickens per replicate. The chickens were housed in three-tiered cages, fed at 8:30 AM daily, and had ad libitum access to clean water via nipple drinkers. Proper ventilation and regular manure removal were maintained in the henhouse. The control group was fed a basal diet, while two experimental groups were designed with different lycopene supplementation levels: the second group (G60) received the basal diet supplemented with 60 mg/kg lycopene, and the third group (G240) with 240 mg/kg lycopene. The feeding trial lasted 37 days, including a 7-day pre-trial period and a 30-day formal trial period. Eggs were collected at 10:00 AM each day.

Table S1 Detailed information of identified metabolites

**Compoundtime**	**Formula**	**KEGG ID**	**Retention Time(time)**	**Acquisition mode**	**Compound type**
Lithocholic acid	C24H40O3	C03990	15.028	Positive	Sterols
L-proline	C5H9NO2	C00148	0.970	Positive	Amino acids
15S-hydroperoxy-11Z,13E eicosadienoic acid	C20H36O4	-	14.799	Negative	Others
L-selenocysteine	C3H7NO2Se	C05688	19.667	Positive	Amino acids
ergosta-3β,5α,6β,25-tetrol	C28H48O4	-	14.835	Positive	Sterols
all-trans-4-Oxoretinoic acid	C27H46O3	C15520	10.376	Positive	Vitamin
L-Valine	C5H11NO2	C00183	0.892	Positive	Amino acids
Adenine	C5H5N5	C00147	3.799	Negative	Vitamin
L-cystathionine	C7H14N2O4S	C02291	11.835	Positive	Amino acids
Glutathionylspermidine	C17H34N6O5S	C05730	11.086	Negative	Peptide
Hydroxyspheroidenone	C41H60O3	C15905	17.835	Negative	Carotenoid
sn-glycero-3-phosphocholine	C8H21NO6P	C00670	0.875	Positive	Others
Succinylbenzoate	C11H10O5	C02730	11.835	Positive	Ketone

Galactitol 1-phosphate	C6H15O9P	C06311	11.804	Negative	Sugar

Table S2 Pathways affected by tebuconazole exposure

Pathway name	Total	hits	-Log(*P*)	FDR	Impact
Cysteine and methionine metabolism	33	1	1.219	0.044	0.179
Arachidonic acid metabolism	41	2	1.138	0.031	0.116
Selenocompound metabolism	18	1	1.403	0.053	0.068
Glycerophospholipid metabolism	36	1	1.062	0.038	0.050

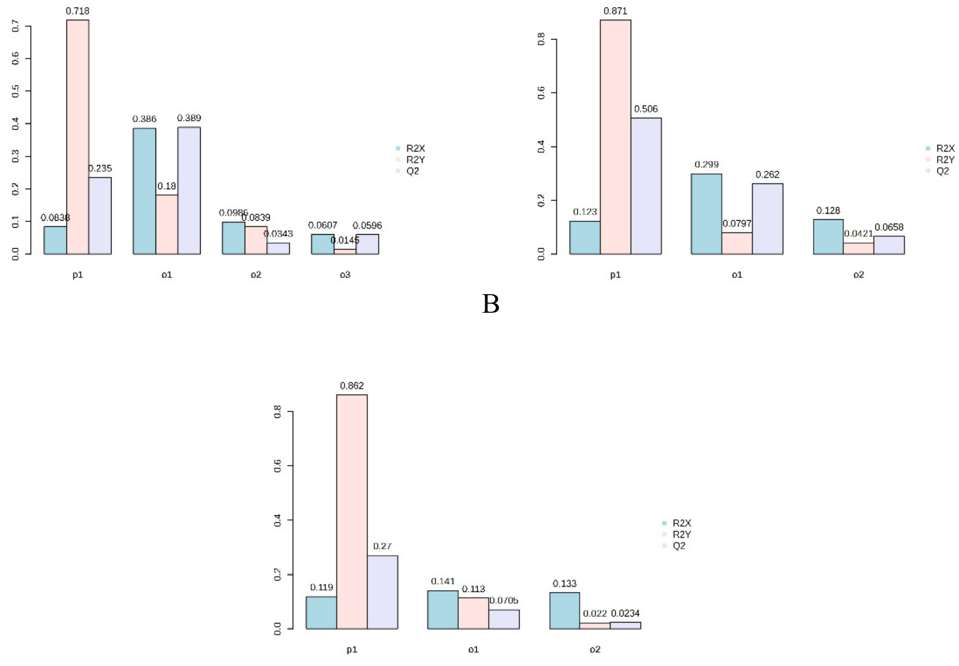

A B C

Fig.S1 Multivariate statistical model performance evaluation for OPLS-DA models (A:G60 vs CK; B:G240 vs CK; C: G240 vs G60; CK, control group; G60, group supplemented with 60 mg lycopene per kg of feed; G240, group supplemented with 240 mg lycopene per kg of feed.)

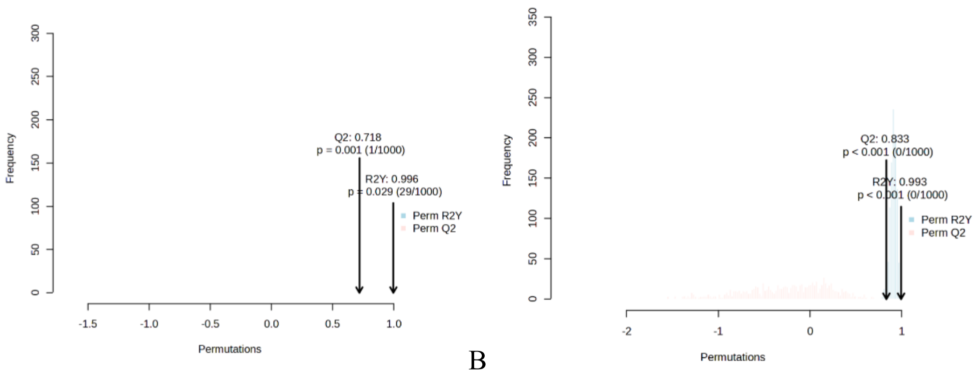

A B

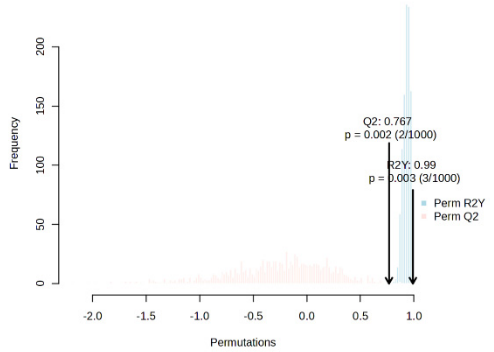

C

Fig.S2 Permutation plot for OPLS-DA models (A:G60 vs CK; B:G240 vs CK; C: G240 vs G60; CK, control group; G60, group supplemented with 60 mg lycopene per kg of feed; G240, group supplemented with 240 mg lycopene per kg of feed.)

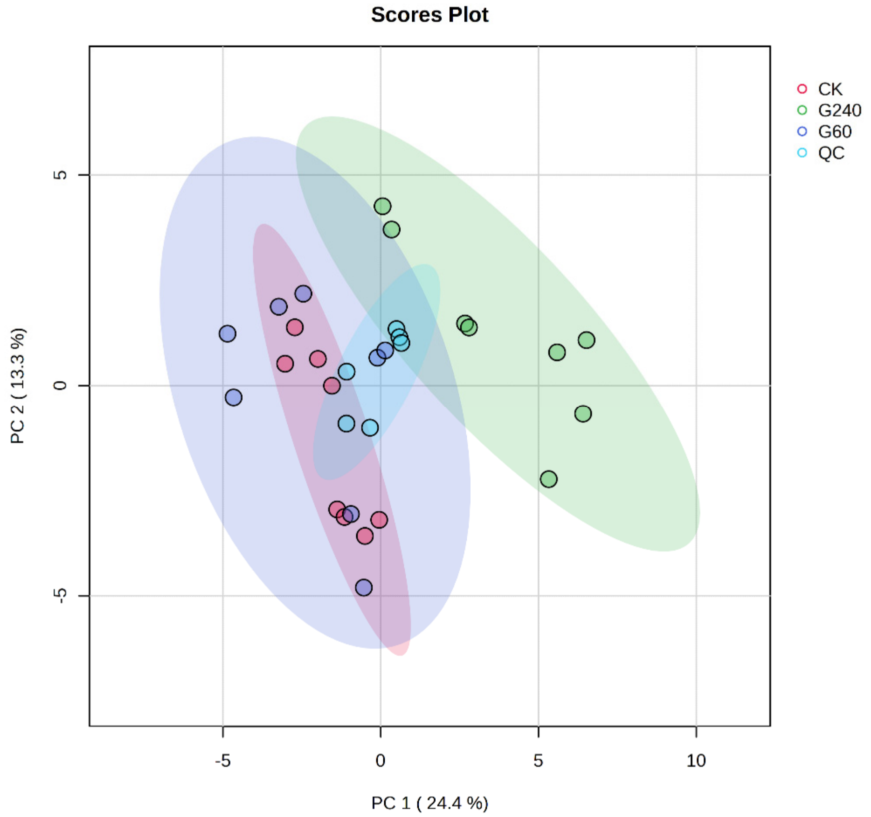

Fig. S3 The PCA score plots based on the lipidomic profiles in egg yolk under different lycopene enrichment(CK, control group; G60, group supplemented with 60 mg lycopene per kg of feed; G240, group supplemented with 240 mg lycopene per kg of feed; QC, quality control sample. CK-1 to CK-6, G60-1 to G60-6, and G240-1 to G240-6 represent the six biological replicates in the CK, G60, and G240 groups, respectively.)

LIthochollc acid

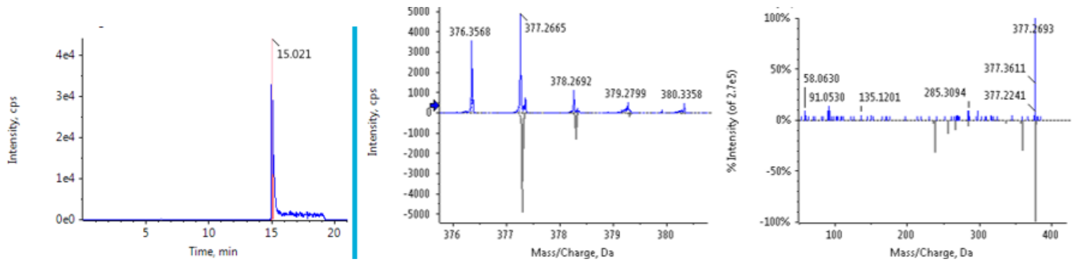

L-proline

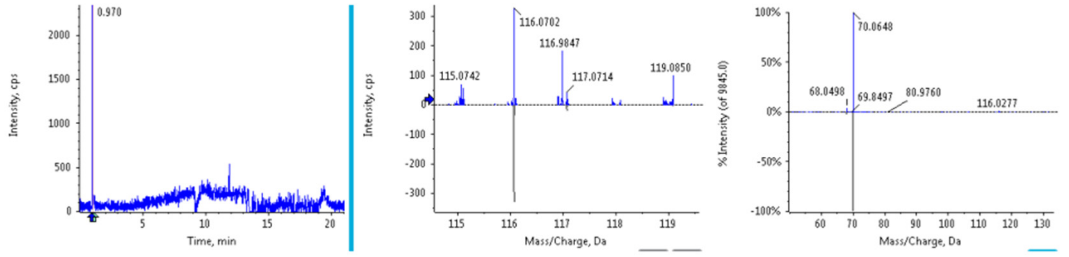

15S-Hydroperoxy-11Z,13E-eIcosadIenoIc acid

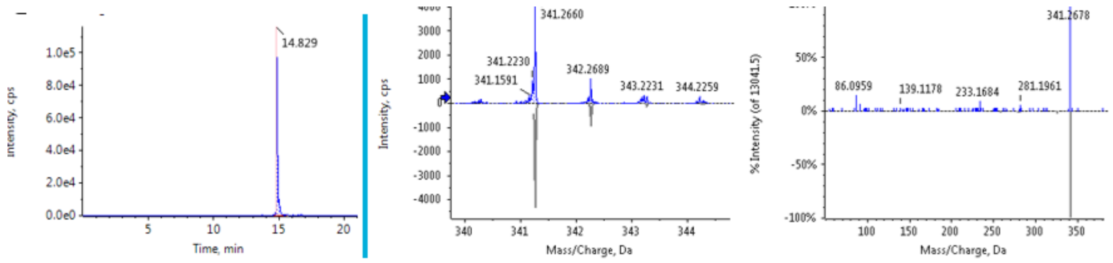

L-selenocysteine

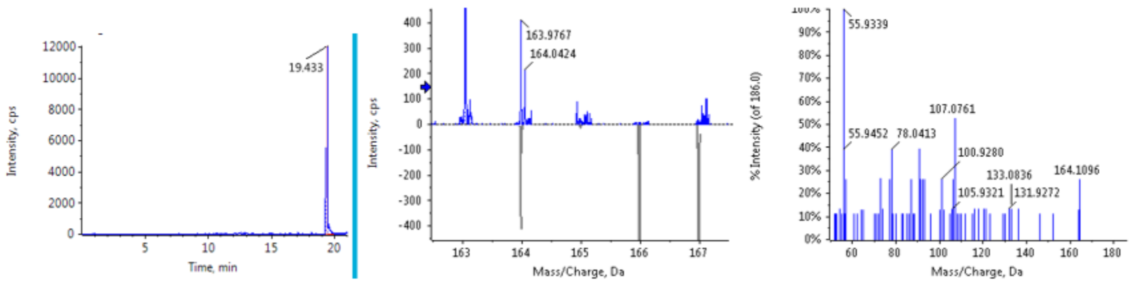

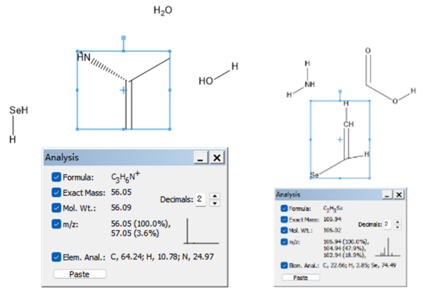

ergosta-3β,5α,6β,25-tetrol

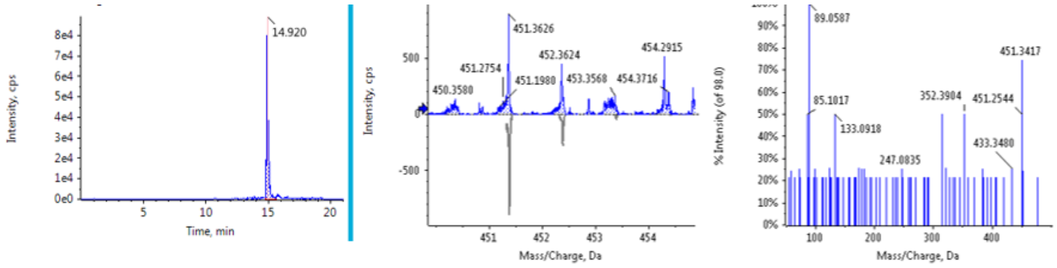

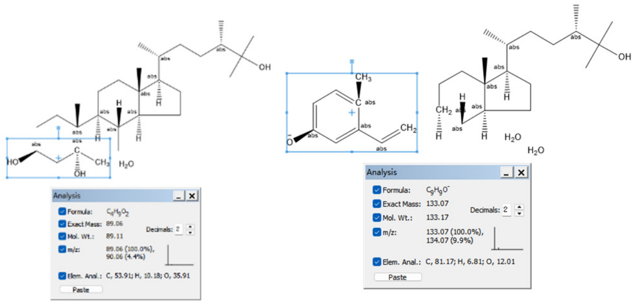

all-trans-4-Oxoretinoic acid

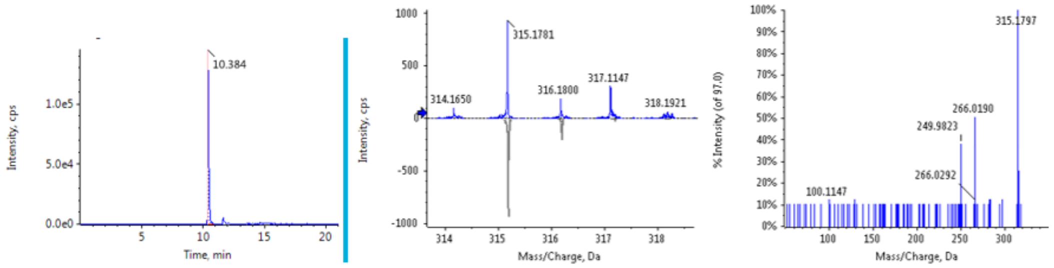

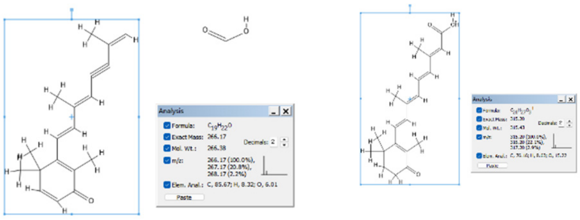

L-valine

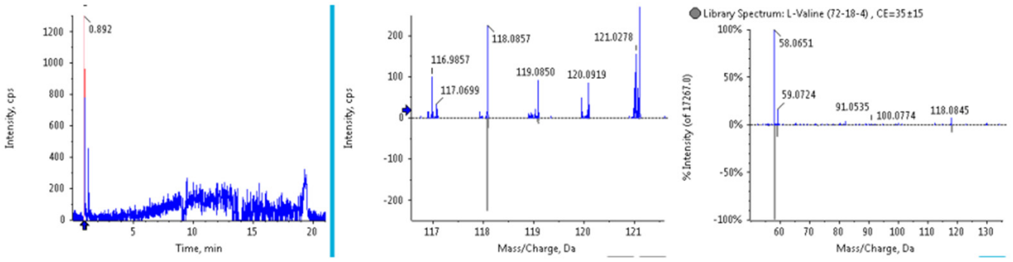

Adenine

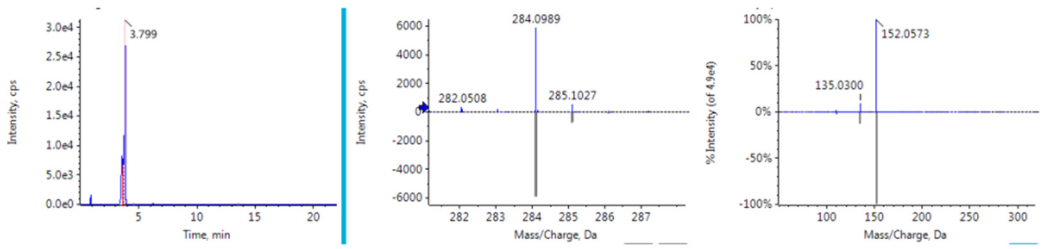

L-cystathionine

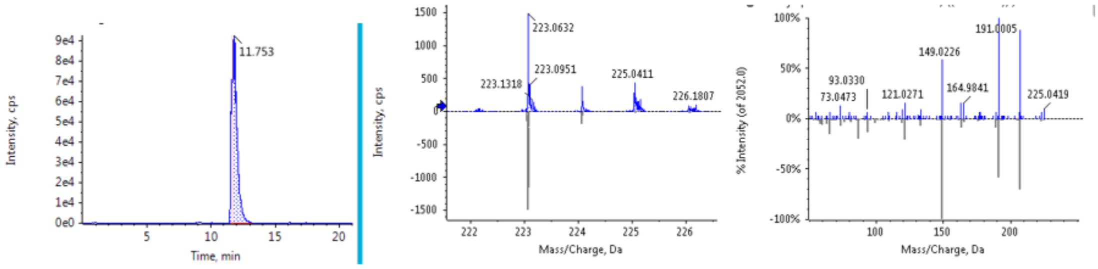

Glutathionylspermidine

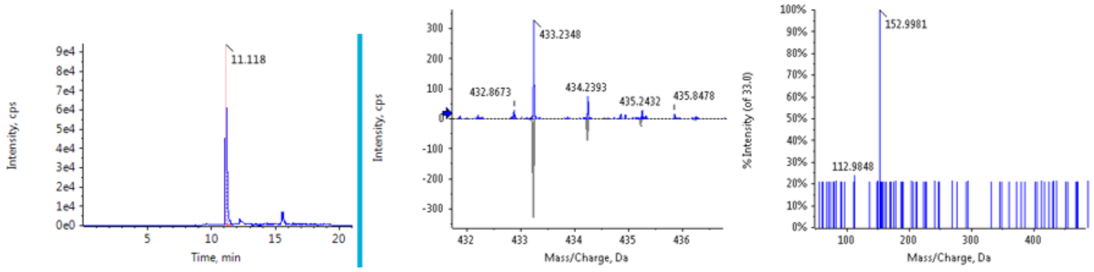

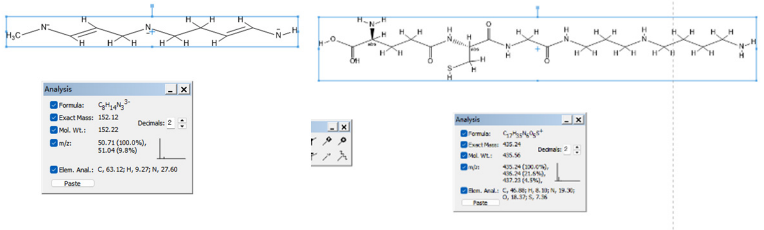

Hydroxyspheroidenone

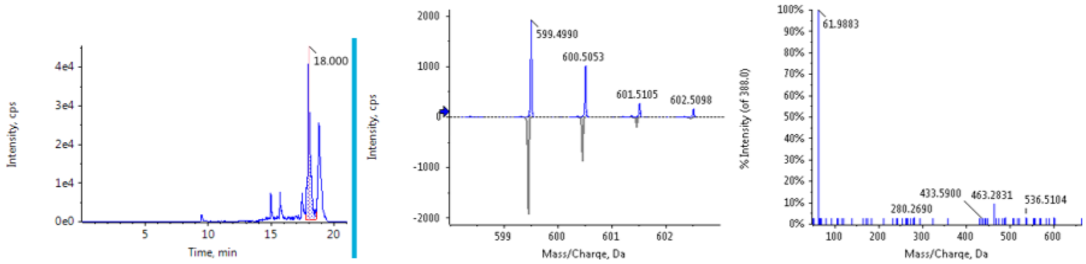

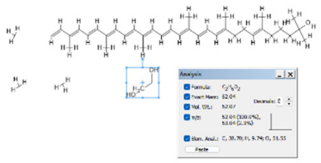

sn-glycero-3-phosphocholine

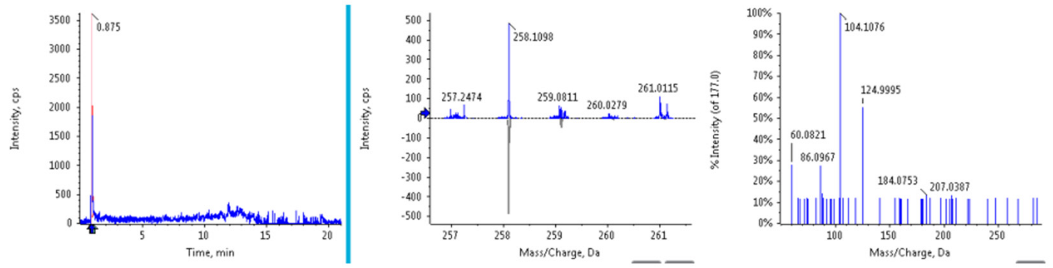

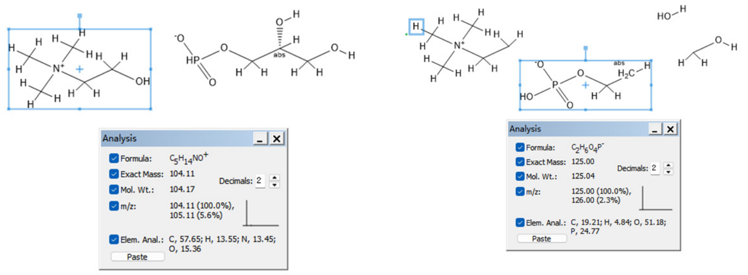

Succinylbenzoate

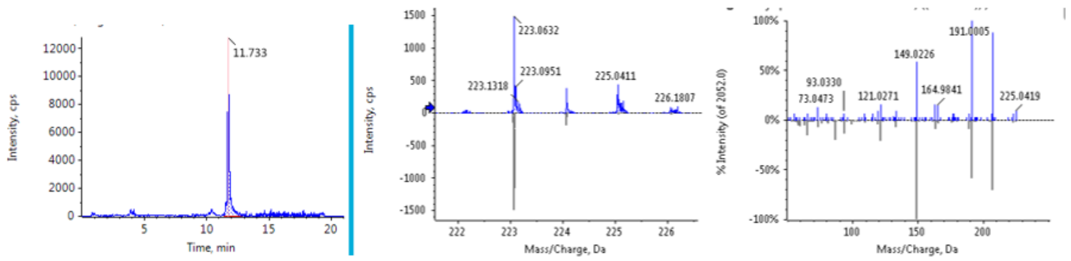

Galactitol 1-phosphate

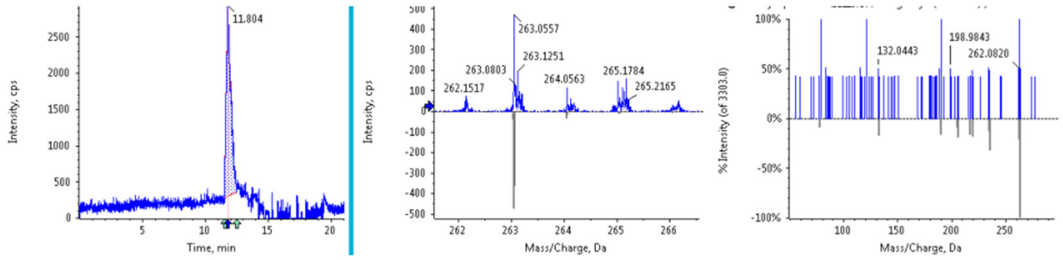

Fig.S4 Extracted ion chromatogram, isotope pattern, fragmentation patterns analyzed with ChemDraw and MS/MS spectrum against library of the identified metabolites

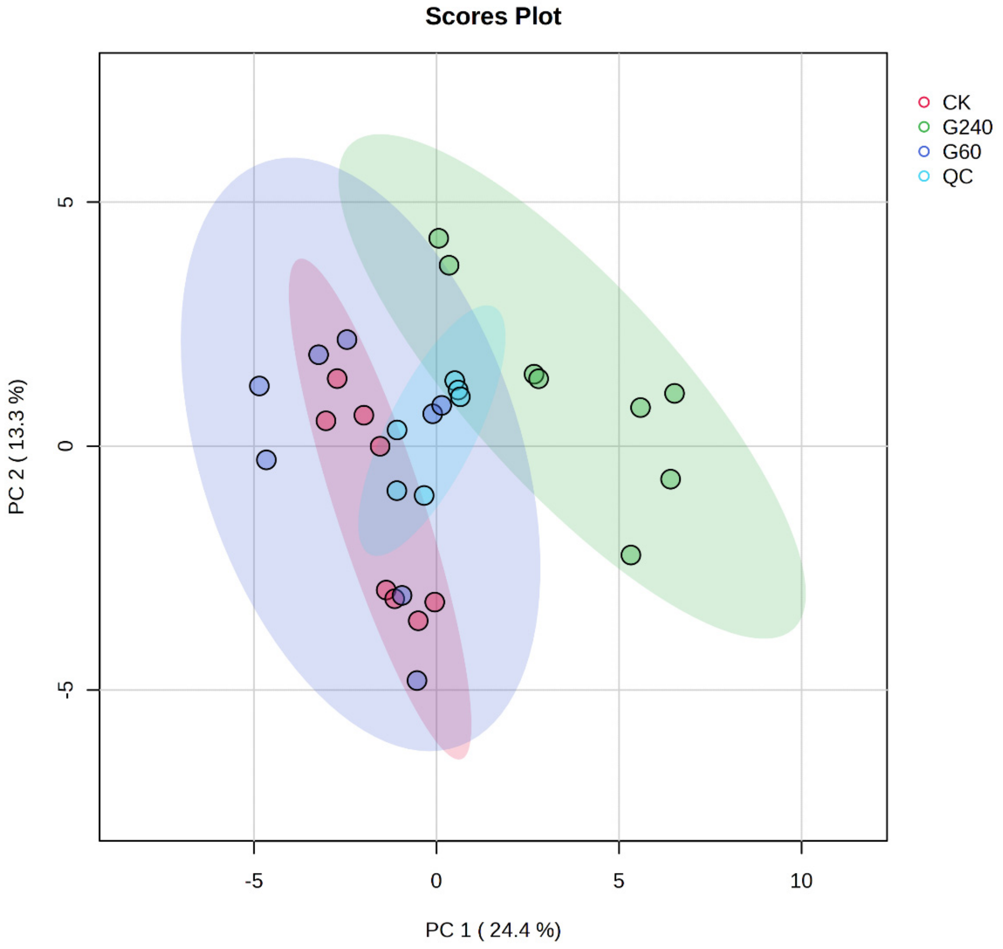

Figure. S5 The PCA score plot based on the lipid profiles of egg yolk under different lycopene enrichment. The colored ellipses represent the 95% confidence regions for each group. (CK, control group; G60, group supplemented with 60 mg lycopene per kg of feed; G240, group supplemented with 240 mg lycopene per kg of feed; QC, quality control sample.)
